# Live‐cell RESOLFT nanoscopy of transgenic *Arabidopsis thaliana*

**DOI:** 10.1002/pld3.261

**Published:** 2020-09-03

**Authors:** Sebastian Schnorrenberg, Hassan Ghareeb, Lars Frahm, Tim Grotjohann, Nickels Jensen, Thomas Teichmann, Stefan W. Hell, Volker Lipka, Stefan Jakobs

**Affiliations:** ^1^ Department of NanoBiophotonics Max Planck Institute for Biophysical Chemistry Göttingen Germany; ^2^ Department of Plant Cell Biology Albrecht‐von‐Haller Institute of Plant Sciences University of Göttingen Göttingen Germany; ^3^ Central Microscopy Facility of the Faculty of Biology and Psychology University of Göttingen Göttingen Germany; ^4^ Clinic of Neurology University Medical Center of Göttingen Göttingen Germany; ^5^ Present address: Department of Plant Biotechnology National Research Centre Cairo Egypt

**Keywords:** fluorescence lifetime gating, live cell imaging, plant, rsEGFP, super‐resolution microscopy

## Abstract

Subdiffraction super‐resolution fluorescence microscopy, or nanoscopy, has seen remarkable developments in the last two decades. Yet, for the visualization of plant cells, nanoscopy is still rarely used. In this study, we established RESOLFT nanoscopy on living green plant tissue. Live‐cell RESOLFT nanoscopy requires and utilizes comparatively low light doses and intensities to overcome the diffraction barrier. We generated a transgenic *Arabidopsis thaliana* plant line expressing the reversibly switchable fluorescent protein rsEGFP2 fused to the mammalian microtubule‐associated protein 4 (MAP4) in order to ubiquitously label the microtubule cytoskeleton. We demonstrate the use of RESOLFT nanoscopy for extended time‐lapse imaging of cortical microtubules in *Arabidopsis* leaf discs. By combining our approach with fluorescence lifetime gating, we were able to acquire live‐cell RESOLFT images even close to chloroplasts, which exhibit very strong autofluorescence. The data demonstrate the feasibility of subdiffraction resolution imaging in transgenic plant material with minimal requirements for sample preparation.

## 1 INTRODUCTION

Super‐resolution microscopy covers a wide range of fluorescence microscopy methods enabling a higher optical resolution than the 200–300 nm attainable with conventional diffraction‐limited microscopy (for comprehensive reviews see (Hell, 2007; Sahl, Hell, & Jakobs, 2017; Sigal, Zhou, & Zhuang, 2018)). Thereby, the term super‐resolution microscopy is typically used for approaches that just extend the resolution by a factor of two to around 100–150 nm, as well as for nanoscopy approaches that tackle the diffraction barrier in a fundamental way, enabling a spatial resolution into the (tens of) nanometer range.

A prominent example of extended‐resolution microscopy is structured illumination microscopy (SIM) (Gustafsson, 2000). SIM microscopy has been successfully used for imaging plant cells (Komis, Samajova, Ovecka, & Samaj, 2015; Schubert, 2017; Shaw, Thoms, & Powers, 2019). It enables fast recordings, albeit at the prize of a limited resolution and the need for computational image reconstruction that may entail the generation of artifacts (Sahl et al., 2016). To reach the sub‐100 nm resolution regime in living cells, genuine fluorescence nanoscopy is mandatory. Although nanoscopy has developed into a standard method for imaging mammalian cells (Sahl et al., 2017), it has so far not been explored to study living green plant tissues and only rarely in nongreen or chemically fixed plant cells (Komis, Novak, Ovecka, Samajova, & Samaj, 2018; Schubert, 2017). Likely reasons for the so far limited use of nanoscopy in plants are aberrations induced by the cell wall, the high autofluorescence of chloroplasts, and concerns on phototoxicity due to the need for relatively high light intensities. Despite these challenges, single‐particle tracking with photoactivated localization microscopy (sptPALM), enabling the visualization of the dynamics of single molecules with nanoscopic resolution, has been reported in various plant systems (Gronnier et al., 2017; Hosy, Martiniere, Choquet, Maurel, & Luu, 2015; Martiniere et al., 2019; Platre et al., 2019).

The wave‐nature of light ultimately confines the minimal size of the volume into which the light in a microscope can be focused. The resolution of conventional fluorescence microscopy is limited because all fluorophores in a diffraction‐limited volume are excited together and therefore fluoresce together. The diffraction barrier can be overcome by driving the fluorophores between two states, typically a fluorescent on‐ and a nonfluorescent off‐state, so that fluorophores residing in a diffraction‐limited volume can be separated (Hell, 2007). Practically, this transient state separation is implemented either by a pattern of light driving this separation, as is the case in the family of coordinate‐targeted nanoscopy, or randomly in space. The latter family of approaches, called coordinate‐stochastic nanoscopy, includes methods such as (f)PALM, STORM, PAINT, and relies on the localization of individual molecules in a wide‐field microscope, and is only rarely used for imaging dynamic structures in living cells (Sigal et al., 2018).

Coordinate‐targeted nanoscopies, with STimulated Emission Depletion (STED) nanoscopy and the related REversible Saturable Optical Linear Fluorescence Transitions (RESOLFT) nanoscopy being the most prominent examples, are scanning approaches. Both methods have been previously used for the imaging of cellular dynamics in animal tissues (Berning, Willig, Steffens, Dibaj, & Hell, 2012; Dreier et al., 2019; Schnorrenberg et al., 2016); to the best of our knowledge they have not been used to image living plant cells or green tissues. For this study, we decided to evaluate RESOLFT nanoscopy (Brakemann et al., 2011; Grotjohann et al., 2011; Hell, Dyba, & Jakobs, 2004; Hofmann, Eggeling, Jakobs, & Hell, 2005) for the imaging of nanoscale structures in cells of intact plant tissues.

RESOLFT nanoscopy harnesses reversibly switchable fluorescent proteins (RSFPs), which can be repeatedly switched by light between a fluorescent on‐ and a nonfluorescent off‐state (Jensen, Jansen, Kamper, & Jakobs, 2020). RSFPs are closely related to conventional fluorescent proteins such as the green fluorescent protein (GFP). The best established protein for RESOLFT is the RSFP rsEGFP2, which has been derived from the well‐known nonswitchable protein EGFP (Grotjohann et al., 2012). rsEGFP2 can be excited to fluoresce by light of 488 nm, and the same wavelength will also switch the protein from the on‐ into the off‐state. Light of 405 nm will switch the protein from the off‐ into the on‐state.

To obtain a diffraction‐unlimited image in beam‐scanning RESOLFT nanoscopy (Grotjohann et al., 2011), a significant number of proteins are first transferred into the on‐state by focused irradiation with light of 405 nm wavelength (Figure S1). Then, a doughnut‐shaped focal pattern of light of 488 nm is used to induce the transition from the on‐ to the off‐state, so that only at its central intensity minimum, the proteins can remain in the fluorescent on‐state. These molecules will be recorded by another regularly focused spot of excitation light at 488 nm wavelength. In order to get a full image, this irradiation scheme is applied at every pixel (Figure S1).

RESOLFT nanoscopy has a number of advantages for live‐cell microscopy. From an optical point of view, it is favorable that beam‐scanning RESOLFT nanoscopy can be combined with confocal detection, so that out‐of‐focus autofluorescence, which is in plant tissues notoriously high, is effectively suppressed. Moreover, RESOLFT nanoscopy can be combined with fluorescence‐lifetime imaging microscopy (FLIM) to further separate the actual fluorescence signal from autofluorescence. Most importantly, RESOLFT nanoscopy requires orders of magnitude lower light doses than all other nanoscopies, including STED, PALM, STORM, and (d)STORM/GSDIM, rendering it particularly suitable for live‐cell recordings (Grotjohann et al., 2012; Wäldchen, Lehmann, Klein, van de Linde, & Sauer, 2015).

In this proof‐of‐concept study, we generated *Arabidopsis thaliana* transgenic plants expressing rsEGFP2 fused to the mammalian microtubule‐associated protein 4 (MAP4), in order to image the microtubule cytoskeleton in living cells. We chose to label microtubules, as the microtubule cytoskeleton is dynamic, ubiquitous, and also found close to highly autofluorescent structures such as the chloroplasts. Hence, this approach allows evaluating the power of the imaging approach for live cell plant recordings. We demonstrate the use of RESOLFT microscopy for time‐lapse imaging in living green leaf tissues with unprecedented resolution. By combining our approach with fluorescence lifetime gating, we were able to acquire live‐cell RESOLFT images even close to highly autofluorescent chloroplasts.

## 2 RESULTS

### 2.1 Generation of rsEGFP2‐MAP4 expressing *Arabidopsis thaliana*

A fusion out of the microtubule binding domain of the mammalian microtubule‐associated protein 4 (MAP4) gene with the green fluorescent protein (GFP) is a powerful tool to highlight cortical microtubules in the model plant *Arabidopsis thaliana* (Marc et al., 1998). As GFP is not suited for RESOLFT nanoscopy, we generated *A. thaliana* plants expressing an rsEGFP2‐MAP4 fusion construct under the transcriptional control of the ubiquitin 10 promoter. These plants also harbored a Basta selectable marker to allow identification of transgenic lines. We screened the fifth leaves of 15 independent Basta‐resistant plant lines from the T1 generation for the presence of an rsEGFP2‐MAP4 fluorescence signal using confocal microscopy. Three, four, and eight transgenic lines showed week, moderate, and high levels of fluorescence signals respectively. We generated homozygous plants from three independent transgenic lines that showed high fluorescence levels. A representative homozygous line of the T4 generation was used for imaging in this study.

**FIGURE 1:**
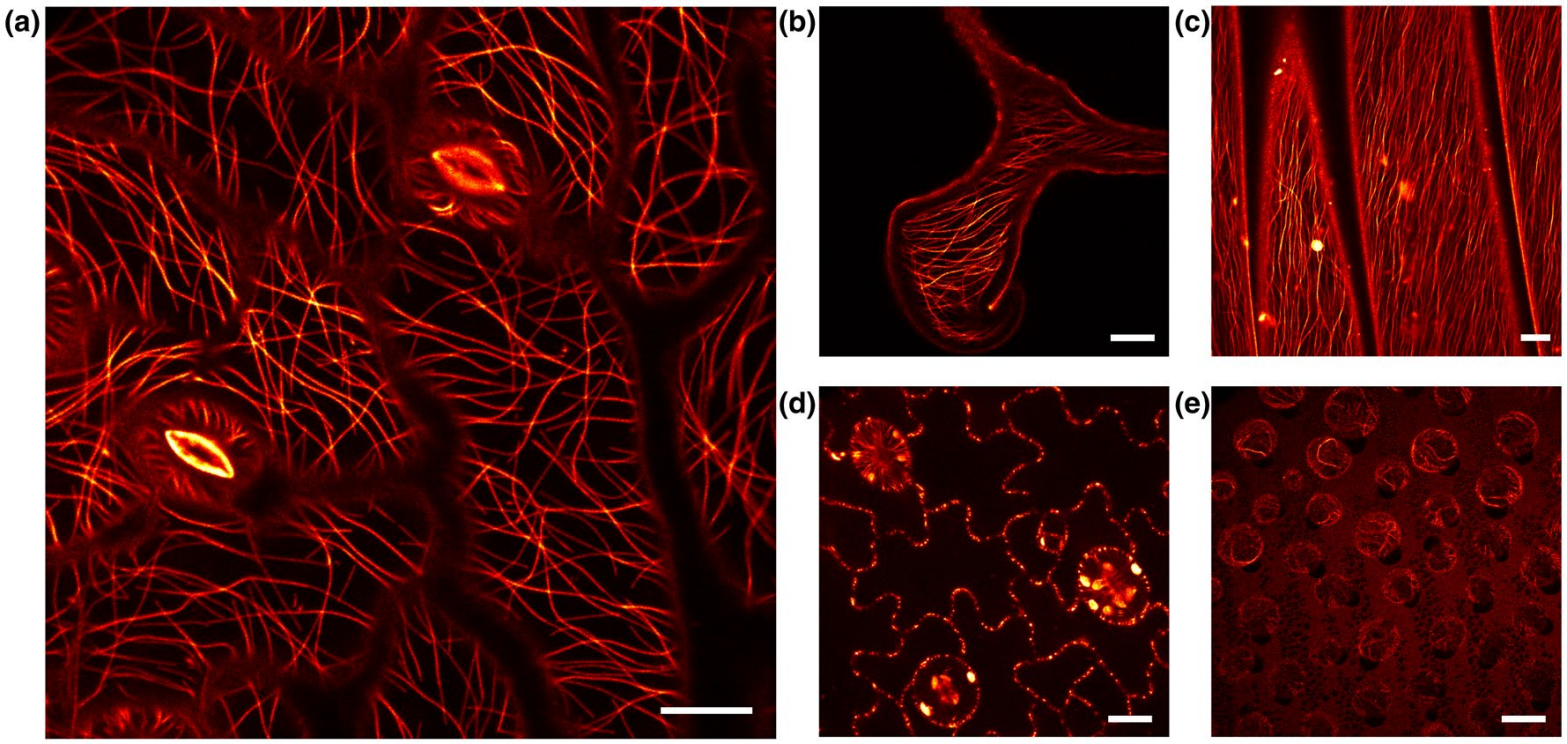
Confocal microscopy of rsEGFP2‐MAP4 labeled tubulin filaments in *A. thaliana*. (a) Abaxial leaf epidermis with stomata cells. (b) Trichome on adaxial epidermis. (c) Petiole. (d) Optical section of abaxial leaf epidermis cells. (e) Sepal cells. Scale bars: 10 µm

First, we investigated the localization of the rsEGFP2‐MAP4 fusion proteins in various tissues. To this end, plant tissue samples were placed in PBS and immediately imaged by diffraction‐limited confocal microscopy. A specific labeling of the microtubule network was visible in cells of the leaf epidermis, the petioles, the sepals, and the petals, as well as in stomata and trichomes (Figure 1). Next to the rsEGFP2 signal, because of their spectrally broad autofluorescence, chloroplasts were brightly fluorescent, concealing the rsEGFP2‐decorated microtubules in their vicinity (Figure 1c,d). Together, the homozygous rsEGFP2‐MAP4 plants were viable and fertile and showed a specific staining of the cortical microtubule network.

### 2.2 Live‐cell RESOLFT‐microscopy of *Arabidopsis thaliana*

**FIGURE 2:**
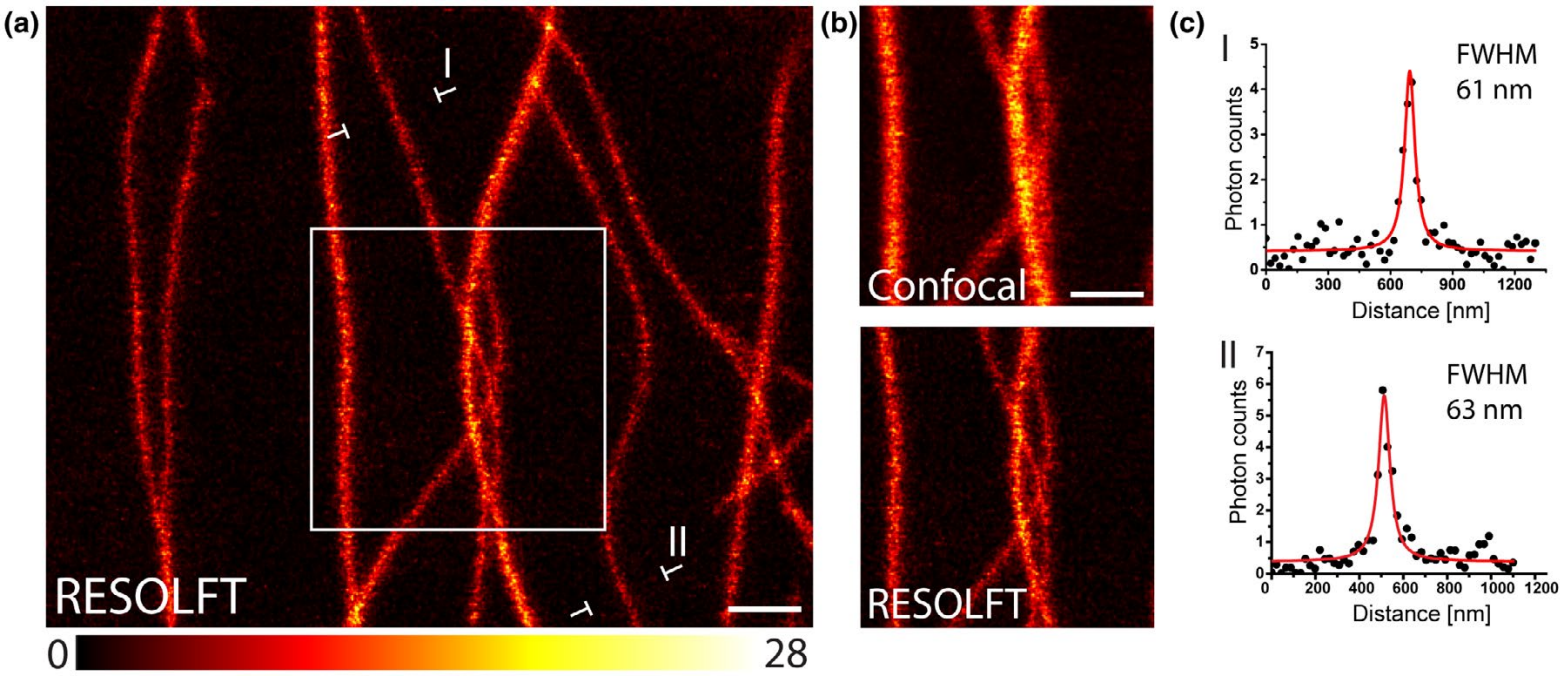
Live‐cell RESOLFT imaging of rsEGFP2‐MAP4 labeled tubulin filaments in leaf epidermis cells of *Arabidopsis thaliana*. (a) RESOLFT image showing 3–4 times resolution enhancement compared to the corresponding diffraction‐limited confocal image. (b) Comparison of confocal and RESOLFT recordings from the area indicated in (a). (c) Line profiles taken at the indicated positions in (a). Each data point represents an average of five adjacent measurements, each separated by 22 nm. The averaged data were fitted with a Lorentzian function (solid red line). The FWHM was determined on the fitted function. Images display raw data without background subtraction. The colormap indicates the actual photon counts. Scale bars: 1 µm

To explore live‐cell RESOLFT nanoscopy in plant tissues, we utilized ~21‐day‐old *A. thaliana* plants expressing rsEGFP2‐MAP4. Leaf discs with a diameter of around 5 mm, taken from the 3rd or 4th rosette leaf, were placed in PBS, covered with a cover glass and directly used for RESOLFT imaging without any additional treatment or further elaborate preparation procedure. We employed a beam‐scanning RESOLFT microscope providing a Gaussian shaped beam of 405 nm to transfer the rsEGFP2 molecules in the fluorescent on‐state, a doughnut of 488 nm light to transfer the peripheral proteins in the non‐fluorescent off‐state and a Gaussian shaped beam of 488 nm to read out fluorescence (Figure S1). Technical details, including the light powers, pixel dwell times, and pixel sizes are given in Table S1. We recorded RESOLFT images of leaf epidermis cells of rsEGFP2‐MAP4 expressing plants (Figure 2a,b; Figure S2). The images display raw data with linear colormaps and no background subtraction. While co‐aligned microtubule filaments frequently could not be resolved in the confocal mode, they could be optically separated in the RESOLFT imaging mode, demonstrating the gain in resolution. In order to quantify the attained resolution, we measured the diameter of the rsEGFP2‐MAP4 decorated microtubules by averaging five adjacent intensity profiles across a single filament at numerous positions in cells. We determined a Full Width at Half Maximum (FWHM) of 60–70 nm (Figure 2c; Figure S2), representing a resolution enhancement of 3–4 fold compared to the resolution attainable with a confocal microscope (~220 nm).

### 2.3 RESOLFT time‐lapse imaging

**FIGURE 3:**
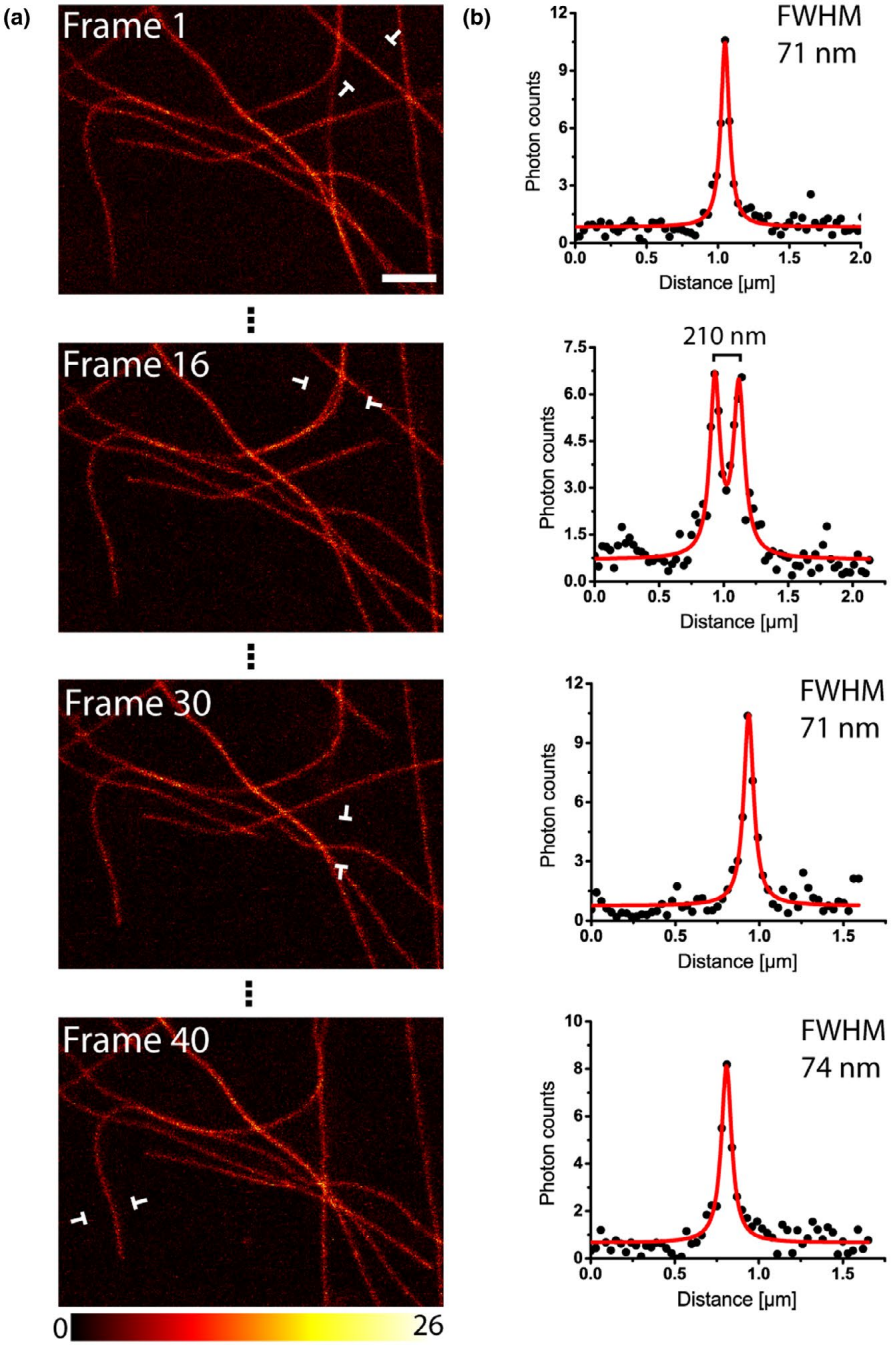
Time‐lapse RESOLFT imaging. (a) Time‐lapse recording of rsEGFP2‐MAP4 labeled microtubules in leaf epidermis cells. Shown are 4 out of 40 images. Imaging was performed continuously. (b) Line profiles taken at the indicated positions in (a). Each data point represents an average of five adjacent measurements, each separated by 30 nm. The averaged data were fitted with a Lorentzian function (solid red line). Images display raw data without background subtraction. For all images the same color coding is used. Scale bar: 2 µm

The microtubule network in epidermis leaf cells exhibits a highly dynamic behavior, with moving, growing, and shrinking microtubule filaments. To capture the dynamic rearrangement of microtubule filaments at high resolution, we recorded 40 consecutive RESOLFT frames within an area of 12.9 µm × 9.7 µm in leaf epidermis cells (Figure 3). We found that during this image series (of in total ~15 min) the photobleaching was negligible. Likewise, at these conditions, the optical resolution, measured as the FWHM of individual filaments, was unaffected during the time series, suggesting a high level of photostability of rsEGFP2 in plant cells (Figure 3). To better visualize the fast movement of single microtubule filaments at high spatial resolution, we adapted the imaging parameters to further shorten the frame acquisition time. Specifically, we reduced the field of view to an area of 9 µm × 6.4 µm and increased the pixel size to 40 nm. This allowed us to capture the grows of single microtubule filaments using RESOLFT time lapse imaging with a time resolution of ~7 s per RESOLFT image frame (Movie S1).

To evaluate the use of rsEGFP2 for extended live‐cell RESOLFT nanoscopy in plant cells further, we continuously imaged a 47 µm^2^ area of cortical microtubules in leaf epidermis cells. We recorded 250 frames in the RESOLFT mode, each with an acquisition time of ~12 s (pixel size: 30 nm) (Figure S3a; Movie S2). During the recording, the cortical network changed substantially, rendering a mere analysis of the overall fluorescence to quantify the photobleaching inappropriate. To address this issue, we normalized in each frame the overall fluorescence of the detected filaments to the length of the microtubules visualized in that frame. This analysis revealed that during the imaging of 250 frames the fluorescence signal decreased by ~30% (Figure S3c,d). We attribute this moderate reduction in fluorescence to the high photostability of rsEGFP2, but also to the fact that we did image only a part of the cell and that during the image sequence new rsEGFP2‐MAP4 molecules presumably appeared in the recorded region.

We also observed that in the leaf cells the cortical microtubules were occasionally so closely co‐aligned that even RESOLFT nanoscopy could not resolve them. The determined average brightness of a single microtubule filament allowed identifying such co‐aligned microtubules (Figure S4; Movie S3).

We conclude that RESOLFT nanoscopy with rsEGFP2 allows extended live‐cell imaging with minimal photobleaching.

### 2.4 Autofluorescence suppression by fluorescence lifetime gating in RESOLFT nanoscopy

**FIGURE 4:**
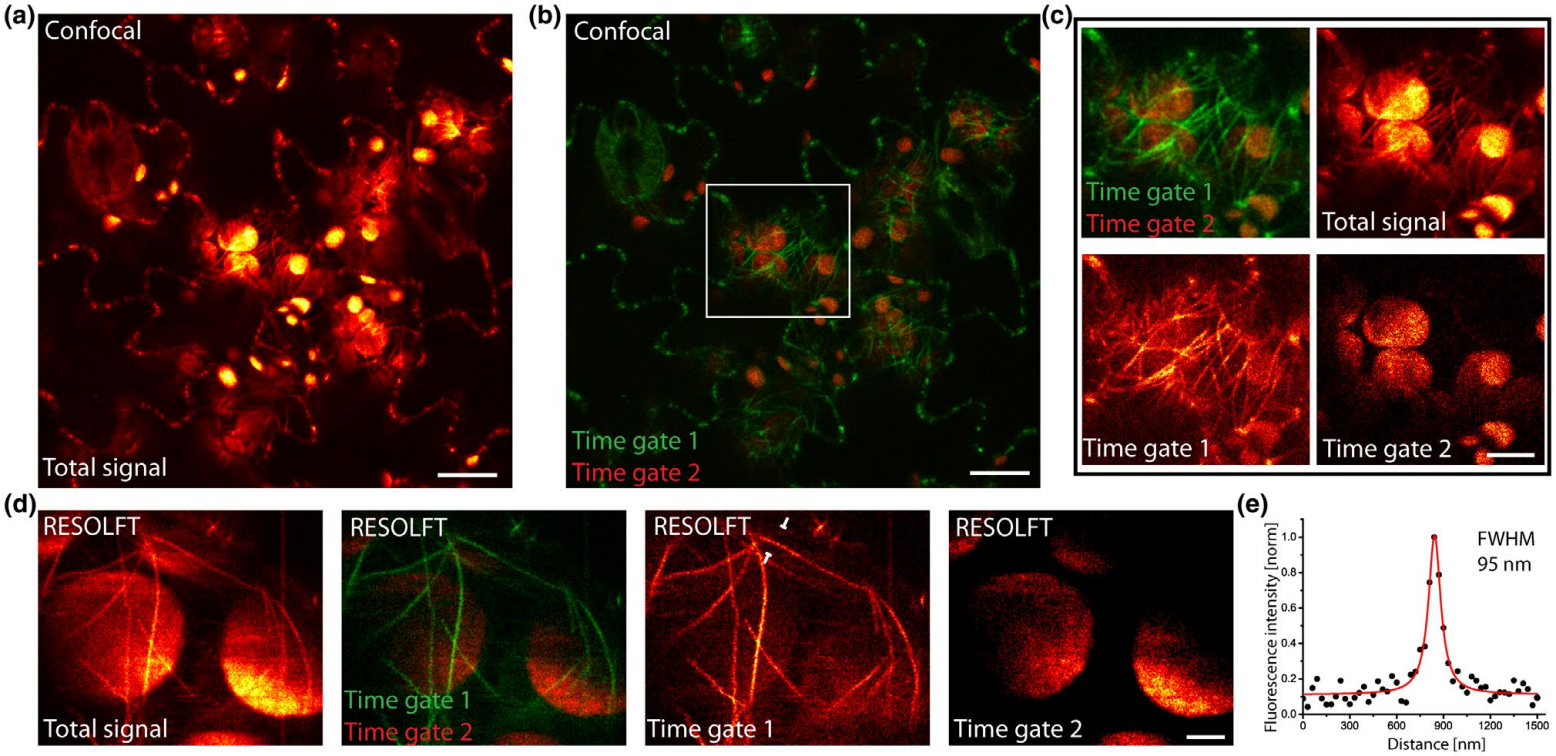
Autofluorescence suppression by fluorescence lifetime gating. (a) Total fluorescence signal (detected at 500–550 nm) of abaxial leaf epidermis cells expressing rsEGFP2‐MAP4. Note the strong autofluorescence of the chloroplasts. (b and c) Fluorescence lifetime gating. The cellular autofluorescence and the rsEGFP2 fluorescence were separated by using distinct fluorescence lifetime gates. (b) Overlay. Autofluorescence shown in red and rsEGFP2 fluorescence in green. (c) Magnification of the area indicated in (b). Shown are the individual detection channels, their overlay, as well as the nongated fluorescence signal (total signal). (d) Fluorescence lifetime gated RESOLFT imaging. Shown are the non‐gated fluorescence signal (total signal), as well as the individual gated channels and an overlay. (e) Line profile of a filament recorded in the RESOLFT mode taken across the position indicated in (d). The data points represent an average of five adjacent measurements. The averaged data were fitted with a Lorentzian function (solid line). The FWHM was determined on the fitted function. Scale bars: 10 µm (a and b), 5 µm (c), and 2 µm (d)

The chloroplast autofluorescence spectrally overlaps with the rsEGFP2 fluorescence; thereby it concealed the rsEGFP2‐MAP4 decorated microtubules located around the organelles (Figure 4a). To address this limitation, we took advantage of the distinct fluorescence lifetimes of the cellular background autofluorescence and of rsEGFP2. The chloroplast autofluorescence, largely due to chlorophyll fluorescence, has a relatively short fluorescence lifetime of ~250 to 300 ps (Krause & Weis, 1991), while rsEGFP2 features a fluorescence lifetime of ~1.6 ns (Testa, D'Este, Urban, Balzarotti, & Hell, 2015). Using fluorescence lifetime gating, the autofluorescence signal of the chloroplast could be separated from the fluorescence of rsEGFP2 when using pulsed excitation and confocal detection (Figure 4a‐c). This approach allowed a clear visualization of rsEGFP2‐MAP4 decorated microtubules near chloroplasts in the diffraction‐limited confocal recording mode.

We next established fluorescence lifetime gated RESOLFT nanoscopy, to explore diffraction‐unlimited imaging of rsEGFP2 labeled structures amidst chlorophyll autofluorescence. Thereby we were able to record individual microtubule filaments immediately adjacent to highly autofluorescent chloroplasts with a resolution of <100 nm (Figure 4d,e).

Taken all together, we demonstrate that low‐light RESOLFT nanoscopy allows to image dynamic nanoscale structures in living highly fluorescent plant tissue requiring only minimal sample preparation.

## 3 DISCUSSION

This study shows that a very simple and straightforward sample preparation approach, involving nothing more than a punched out leaf disk immersed in buffer on a microscope slide, is sufficient for RESOLFT nanoscopy. The only major requirement for successful RESOLFT imaging was that the plant cells expressed a fusion protein with an RSFP such as rsEGFP2 at sufficient levels. We demonstrate recordings of microtubule dynamics within living tissues and, by separating the rsEGFP2 fluorescence signals from the chlorophyll autofluorescence, we were even able to record nanoscale images close to the chloroplast.

Most previous super‐resolutions applications in plant cells relied on SIM, enabling a resolution of 100–120 nm (for comprehensive reviews, see Komis et al., 2015, 2018; Schubert, 2017; Shaw et al., 2019). Yet, the unfavorable refractive index and the birefringence of the plant cell walls provide a significant challenge for SIM approaches, and only a limited number of studies demonstrating SIM in living green tissue have been published. Nanoscopy has been used even more rarely in plant biology. The few examples include the analysis of cellulose microfibrils using TIRF‐dSTORM in onion cells (Liesche, Ziomkiewicz, & Schulz, 2013), of cortical microtubules in chemically fixed root cells using STORM (Dong, Yang, Zhu, Bassham, & Fang, 2015), as well as PALM of perinuclear actin in BY‐2 cells (nongreen suspension cells derived from tobacco) (Durst et al., 2014). STED nanoscopy has been used in chemically fixed cells (Chumova et al., 2018; Demir et al., 2013) and we are aware of only two studies that report its use in living root cells (Kleine‐Vehn et al., 2011; Molines et al., 2018).

To our knowledge, not a single study reported so far on the use of nanoscopy in living green plant tissue; RESOLFT nanoscopy has been used neither in fixed nor in living plant cells. Presumably, the fact that plant tissues contain numerous highly absorbing components, including the cell walls and the chloroplasts, raised concerns on the usability of RESOLFT nanoscopy on plants. A further notorious concern in nanoscopy is phototoxicity due to the light intensities required to overcome the diffraction barrier (Wäldchen et al., 2015). However, in RESOLFT nanoscopy, the light intensities applied are in the same range as usually used for confocal scanning fluorescence microscopy, a technique that has abundantly been used for plant cell recordings (Grotjohann et al., 2012). This study demonstrates that RESOLFT imaging in living intact plant tissue is possible.

When applied on large fields of view, beam‐scanning RESOLFT nanoscopy is a relatively slow approach, and therefore is not necessarily able to capture fast inner‐cellular movements such as the motion of vesicles across a cell. Still, the imaging parameters, including the resolution, the signal‐to‐noise ratio, or the imaging speed can be adapted, within certain limits, to the requirement of the sample. With the ongoing development of RSFPs, and parallelized or adaptive imaging schemes (Chmyrov et al., 2013; Dreier et al., 2019), we predict that the framerate of time lapse RESOLFT imaging will be further increased in the future.

## 4 METHODS AND MATERIALS

### 4.1 Plant materials and growth conditions

Seeds of *Arabidopsis thaliana* ecotype Col‐0 were sown onto 8 cm^2^ pots and vernalized at 4°C for three days. For *Agrobacterium*‐mediated transformation, the plants were grown in a climate chamber (Johnson Controls, USA) under long day conditions (16/8 hr) with 150 μmol·m^−2^·s^−1^ at 22/18°C. For microscopy experiments, the transgenic plants were grown under short day conditions (8/16 hr) with 130 μmol·m^−2^·s^−1^ at 22/18°C.

### 4.2 Plasmid construction

To generate the microtubule marker fusion construct rsEGFP2‐MAP4, the binary vector pC2 that confers *in planta* Basta resistance (Ghareeb, Laukamm, & Lipka, 2016) was used. The UBQ10 promoter was integrated into pC2 via the RsrII restriction sites and the resulting plasmid was linearized with the BamHI and SpeI restriction sites. The rsEGFP2 and MAP4 were generated by PCR amplification from pMD‐Vim‐rsEGFP2 (Grotjohann et al., 2012) and pC3 (Ghareeb et al., 2016) using the oligonucleotide primer pairs CTGATTAACAGCGGTCCGGGATCCATGGTGAGCAAGGGCGAGG and CATCACCGCGTGCTTGTACAGCTCGTCCATGC, and ACAAGCACGCGGTGATGTCCCGGCAAGAAGAAGCAAAG and GGTGATTTTTGCGGACTCTAGACTAGTTTAACCTCCTGCAGGAAAGTGGC, respectively, and then ligated with the linearized vector by Gibson Assembly kit (NEB, Hitchin, UK, to construct the expression vector pUBQ10‐rsEGFP2‐MAP4.

### 4.3 *Arabidopsis* transformation

*Arabidopsis* transformation was performed according to the floral dip method (Clough & Bent, 1998). The pUBQ10‐rsEGFP2‐MAP4 plasmid was transformed into the *Agrobacterium tumefaciens* strain GV3101 pMP90RK. The resulting *Agrobacterium* strain was inoculated in LB liquid medium supplemented with 50 mg/L rifampicin, 30 mg/L gentamicin, 50 mg/L kanamycin, 50 mg/L carbenicillin, and grown for two days at 28°C. The suspension culture was centrifuged at 4,000 g for 20 min and then resuspended in infiltration medium (0.5x MS, 5% sucrose, and 0.015% Silwet L‐77) that was used for floral dipping. Positive transformants were selected on soil by spraying seedlings of the T1 generation with 0.1% BASTA (Bayer CropScience). Plants of the T4 generation were used for microscopy.

### 4.4 Sample preparation

For imaging of leaf epidermis cells, small disks of the leaf were punched out using a dedicated device. These disks were placed in a drop of PBS, covered with a coverslip (Menzel 22 × 40 mm, 1.5H) and sealed using nontoxic silicone (Picodent, Wipperfuerth, Germany).

### 4.5 RESOLFT microscopy

RESOLFT nanoscopy was performed using a modified 1C RESOLFT QUAD Scanning microscope (Abberior Instruments, Göttingen, Germany) equipped with an Olympus UPLSAPO 1.4 NA 100x oil objective and an 1.2 NA 60 x water objective. For RESOLFT imaging of rsEGFP2‐MAP4, at each scanning position the following illumination scheme was applied: First, rsEGFP2 proteins in the focal spot were switched to the on‐state with light of 405 nm. Second, rsEGFP2 proteins in the periphery of the focal spot were transferred to the off‐state using a 488 nm doughnut‐shaped beam. Third, the on‐state fluorophores at the center of the spot were excited with a Gaussian shaped beam of 488 nm light and fluorescence was detected in the GFP channel at 500–550 nm.

For fluorescence lifetime gated confocal and RESOLFT imaging, the microscope was equipped with a pulsed 488 nm laser with a 40 Mhz repetition rate. Additionally, a MPD (Micro Photon Device, Italy) APD with fast timing option (<50 ps FWHM timing resolution) was used as a detector. The fluorescence detection windows were gated and set in the Imspector‐software to 0–312.5 ps (time gate 2) and 2.656–16.71 ns (time gate 1). These settings reduced the total fluorescence signal of rsEGFP2 in the GFP detection channel, since the main peak of the fluorescence was not detected. However, these time windows showed the lowest cross‐talk between the autofluorescence of the chlorophyll and the rsEGFP2 fluorescence signal.

The RESOLFT images were recorded with or without line accumulation depending on the signal intensity. A detailed listing of the laser powers, switching times, line accumulations, and scanning step sizes of all RESOLFT images shown are provided in Table S1.

### 4.6 Data analysis and image processing

All fluorescence images presented in this work are raw data, except where indicated, smoothing by convolution with a 1.2 pixel wide Gaussian using Imspector software was performed. For determination of the FWHM of single microtubule filaments, five adjacent line profiles of raw data images were averaged and fitted (Lorentz‐fit) using Origin 9.1 software.

### 4.7 Filament detection and counting

To detect the tubulin filaments in the image, a ridge detection algorithm based on the calculation of the major eigenvalue of the Hessian Matrix at each pixel of the image was used. In the resulting image of ridges, local maximal lines were found by applying a filter returning one pixel if a pixel was among the three largest pixels of its eight neighbors. The filament brightness was estimated from the smoothed image at the positions of the detected filaments. The median of the brightness values was used to estimate the single filament brightness. The number of filaments was found by dividing the image by the single filament brightness and rounding the result. All procedures were implemented in Matlab.

## CONFLICT OF INTEREST

The authors declare no conflict of interest.

## Supporting information

Fig S1

Fig S2

Fig S3

Fig S4

Table S1

Video S1

Video S2

Video S3
