# Supplementary figures and images for: Live‐cell RESOLFT nanoscopy of transgenic Arabidopsis thaliana
Source: Plant Direct. 2020 Sep 3;4(9):e00261. doi: 10.1002/pld3.261 (PMC7507094; doi:10.1002/pld3.261)

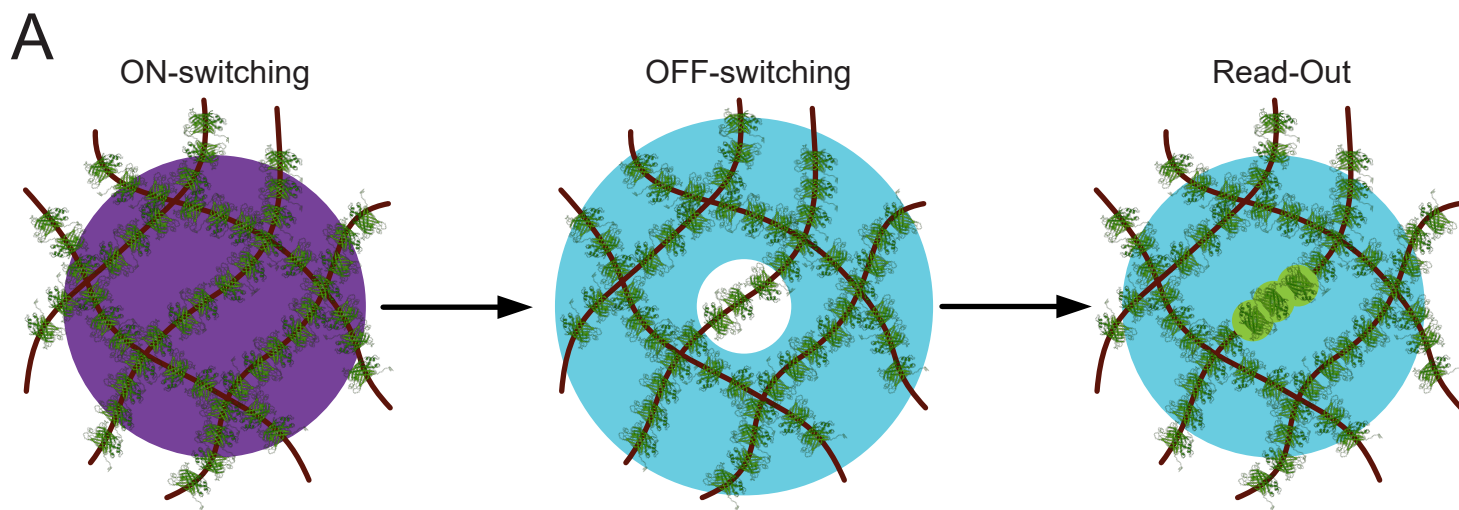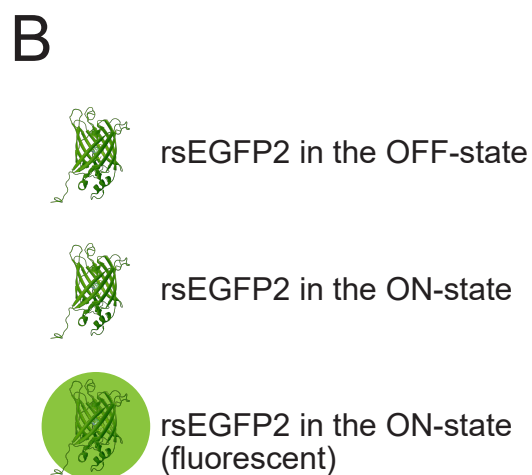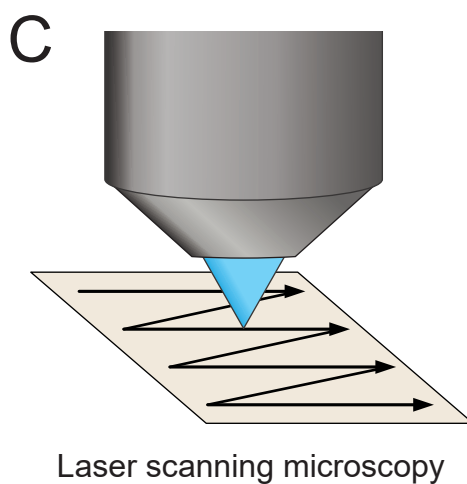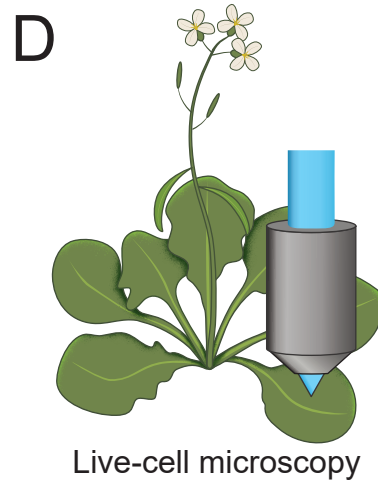

Supplement: Supplementary file 1 — Fig S1 [file PLD3-4-e00261-s001.pdf]

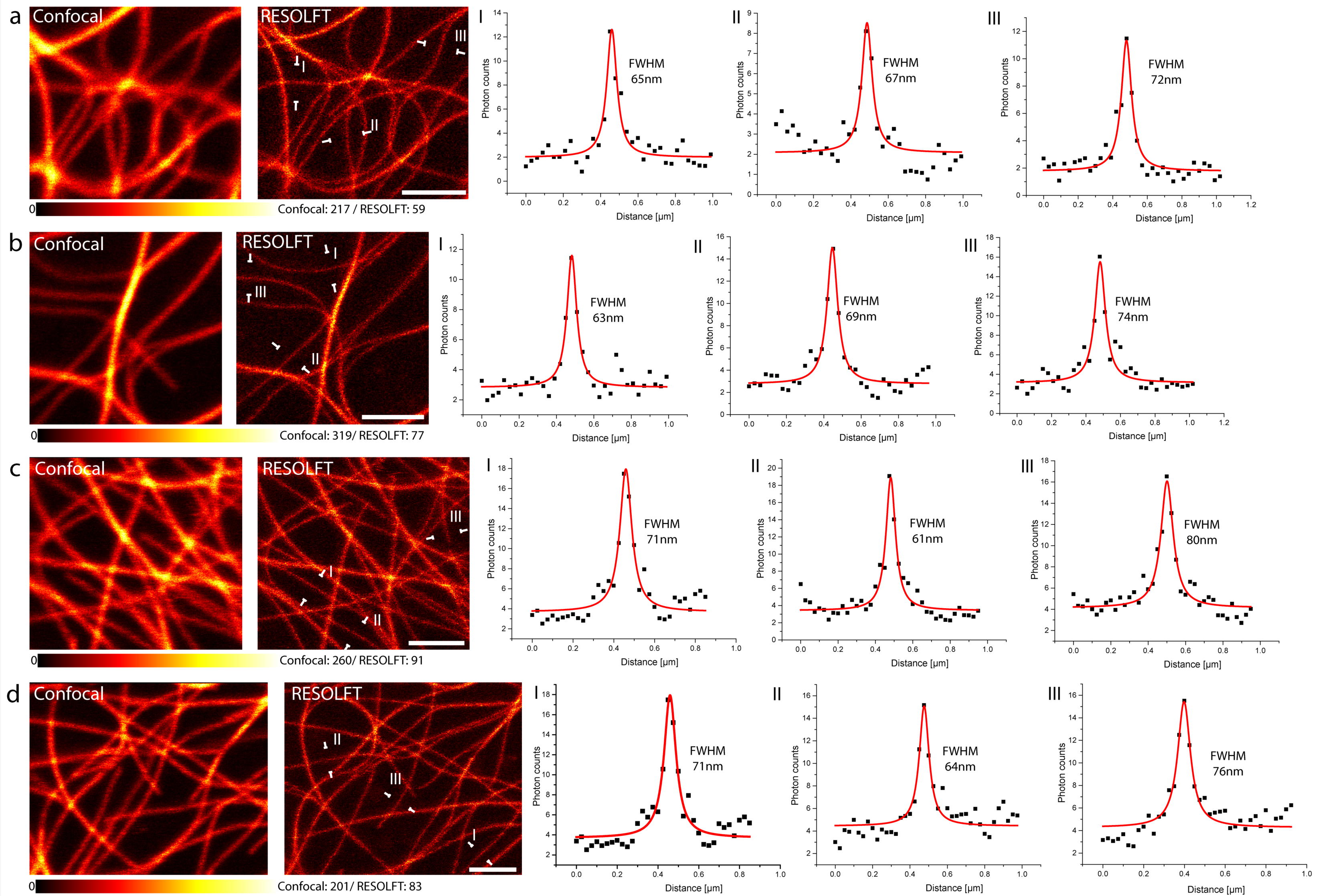

Supplement: Supplementary file 2 — Fig S2 [file PLD3-4-e00261-s002.pdf]

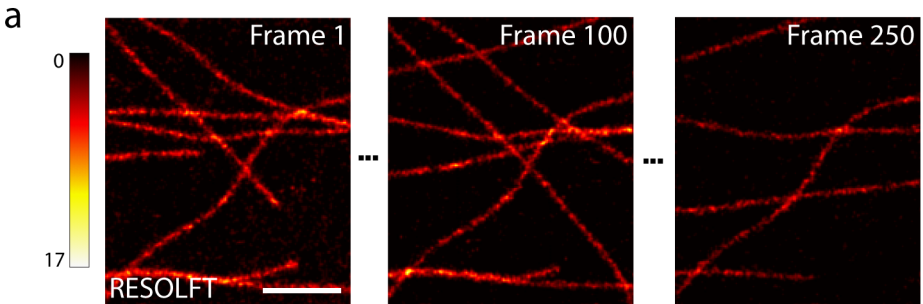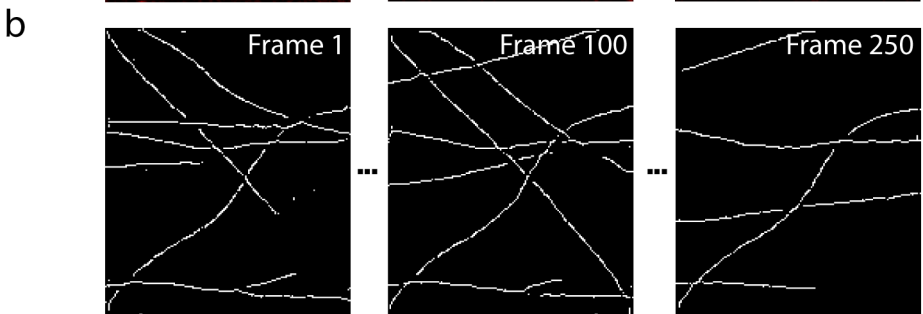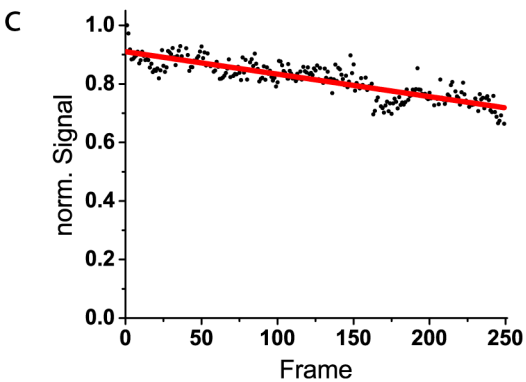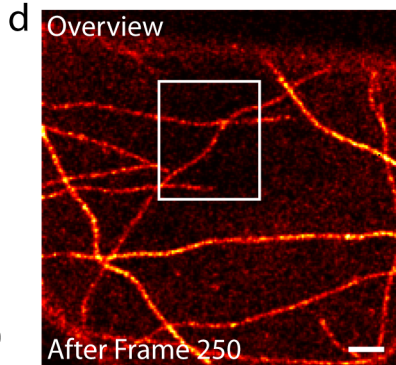

Supplement: Supplementary file 3 — Fig S3 [file PLD3-4-e00261-s003.pdf]

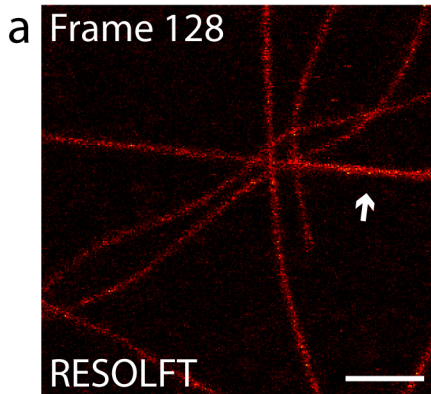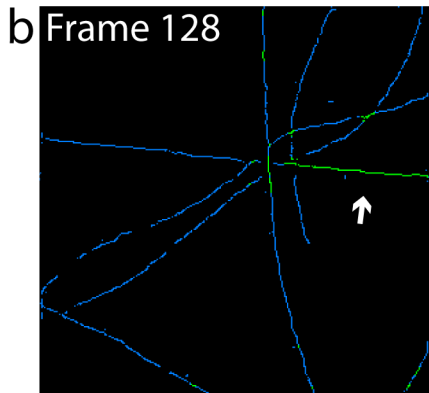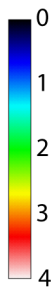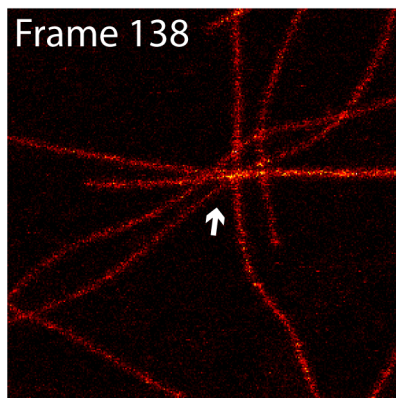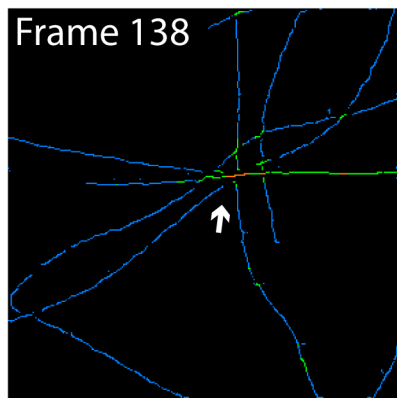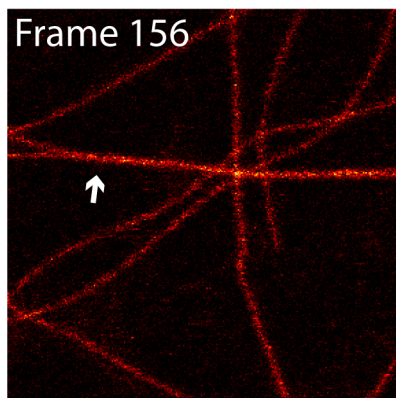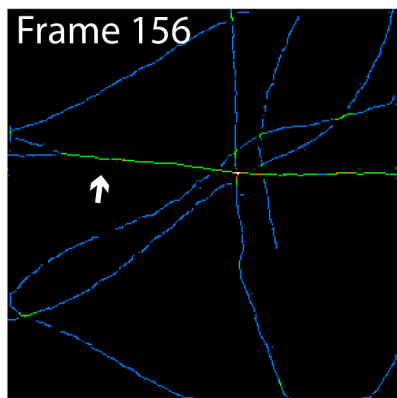

Supplement: Supplementary file 4 — Fig S4 [file PLD3-4-e00261-s004.pdf]
