# Supplementary material for: Live‐cell RESOLFT nanoscopy of transgenic Arabidopsis thaliana
Source: Plant Direct. 2020 Sep 3;4(9):e00261. doi: 10.1002/pld3.261 (PMC7507094; doi:10.1002/pld3.261)
Supplement: Supplementary file 5 — Table S1 [file PLD3-4-e00261-s005.pdf]

RESOLFT-image	405 nm on switching		488 nm off switching beam		488 nm readout		Pixel size xy [nm]	Line accumulation
	Power [μW]	Illumination time [μs]	Power [μW]	Illumination time [μs]	Power [μW]	Illumination time [μs]		
Fig. 2a	3.3	20	8.3	480	18	30	22	1
Fig. 3a	3	12	6	120	11	30	30	1
Fig. 4d	3.7	20	40	280	1	100	30	4
Suppl. Fig. S2a	2.4	20	25	480	9.4	40	30	3
Suppl. Fig. S2b	2.3	20	21	500	9.5	40	30	3
Suppl. Fig. S2c	2.3	20	20	540	9	40	25	2
Suppl. Fig. S2d	2.5	20	35	480	8	40	25	2
Suppl. Fig. S3a	3.7	20	25	340	23	30	40	1
Suppl. Fig. S4a	3.5	12	21	190	16	40	35	1
Suppl. Movie S1	11	10	36	150	12	30	40	1
Suppl. Movie S2	3.7	20	25	340	23	30	40	1
Suppl. Movie S3	3.5	12	21	190	16	40	35	1
